# Investigation of the Influence of Alloying Elements Ni, Cr, Co and Mo on the Crystallization Process, Phase Composition and Corrosion Resistance of AlSi25Cu4Cr and AlSi25Cu5Cr Alloys

**DOI:** 10.3390/ma18040907

**Published:** 2025-02-19

**Authors:** Boyan Dochev, Desislava Dimova, Karel Trojan, Jiří Čapek, Kalina Kamarska, Bozhana Chuchulska

**Affiliations:** 1 Department of Mechanical Engineering and Technologies, Faculty of Mechanical Engineering, Technical University of Sofia, Branch Plovdiv, 4000 Plovdiv, Bulgaria; desislava608738@gmail.com (D.D.); kamarska@tu-plovdiv.bg (K.K.); 2 Faculty of Nuclear Sciences and Physical Engineering, Czech Technical University in Prague, Trojanova 13, 120 00 Prague, Czech Republic; karel.trojan@fjfi.cvut.cz (K.T.); jiri.capek@fjfi.cvut.cz (J.Č.); 3 Department of Prosthetic Dental Medicine, Faculty of Dental Medicine, Medical University of Plovdiv, 4000 Plovdiv, Bulgaria

**Keywords:** hypereutectic aluminum–silicon alloys, thermal analysis, phase composition, corrosion resistance

## Abstract

To increase the mechanical and improve the operational properties of the AlSi25Cu4Cr and AlSi25Cu5Cr alloys, combinations of the alloying elements Ni, Co and Mo were used. The AlSi25Cu4Cr alloy was additionally alloyed with both Ni and Mo and Ni, Co and Mo, and the AlSi25Cu5Cr alloy was alloyed with Co and Mo in different concentrations. The dental alloys “wiron light” and “wironit” were used to introduce the elements Ni, Co, Mo, as well as additional amounts of Cr into the composition of the base compositions. The thermal analysis recorded a decrease in the liquidus and solidus temperatures of the base alloys, as well as a narrowing of their crystallization temperature range as a result of the added alloying elements. The influence of the used chemical elements on the phase composition of the alloys was established by X-ray diffraction. The elements Cr and Mo do not form secondary strengthening phases but dissolve in the α-solid solution. The results of the corrosion tests conducted in 1 M HCl solution and 1 M H_2_SO_4_ solution for 336 h and 504 h show that the elements Ni, Co and Mo improve the corrosion resistance of the alloys.

## 1. Introduction

Aluminum alloys have found wide application in mechanical engineering due to their good combination of mechanical, physical and operational properties. These properties directly depend on the microstructure of the alloys, which in turn depends on the alloying elements and modifiers used. Alloys from the aluminum–silicon system belong to the foundry aluminum alloys. Hypoeutectic and eutectic compositions are characterized by good technological, mechanical and operational properties, while hypereutectic alloys from the system have poorer technological properties but have a lower coefficient of thermal expansion upon heating and better wear resistance compared to other alloys from the system [1,2]. This is the reason why hypereutectic alloys are mainly used for the production of parts operating at elevated temperatures in friction pairs such as pistons for internal combustion engines [3,4].

The influence of some of the alloying elements used to influence the properties of aluminum alloys is presented in [5,6]. The main alloying elements used in the production of piston aluminum–silicon alloys are Si, Mg, Cu and Ni because they have a positive influence on the properties of the alloys after heat treatment [3,4]. The use of Mg in alloys alloyed with silicon leads to the formation of the Mg_2_Si hardening phase and its participation in other intermetallics after T6 thermal treatment, which in turn increases the strength indicators of the alloys but decreases the plastic ones [7,8,9,10].The addition of Cu to aluminum alloys leads to an increase in their mechanical properties [11,12,13,14,15] due to the fact that, after heat treatment, the CuAl_2_ phase is formed and separated. The addition of nickel (Ni) to aluminum alloys leads to an increase in mechanical strength, wear resistance and even plasticity due to the strengthening of the solid solution after heat treatment [16,17,18]; it also has a positive effect on the corrosion resistance of the alloys [19]. When the nickel content in the composition of the alloys increases (above 1 wt%), agglomeration of Ni-rich intermetallics is observed, which in turn is the cause of a decrease in the strength characteristics of the alloys [20].

The presence of iron in the composition of aluminum alloys is undesirable due to the formation of intermetallic phases that deteriorate both the technological and mechanical properties of the alloys. The negative effect and the different morphologies of iron-containing phases are presented in [21]. To eliminate the harmful effect of Fe-rich phases, the elements manganese (Mn), chromium (Cr), cobalt (Co), beryllium (Be), molybdenum (Mo), nickel (Ni), vanadium (V), tungsten (W), strontium (Sr), as well as the rare earth elements cerium (Ce), lanthanum (La), neodymium (Ne) and yttrium (Y) are added to the alloys [21,22]. Elements with a high melting point, such as Cr, Mo, V and W, are rarely used in Al-Si alloys due to their limited solubility in aluminum, but, on the other hand, they can be useful for strengthening the alloys after heat treatment. These elements can form intermetallic phases that make the alloy brittle, especially at lower cooling rates during casting. Therefore, their addition is not recommended in alloys for sand or ceramic casting. At higher cooling rates (casting in molds), a saturated α-solid solution is obtained and the mechanical properties of the alloys are improved [23]. The precise dosage of the amounts of the indicated elements is of utmost importance. As a general rule, a minimum Mn:Fe ratio of 0.5 is recommended, especially when the iron content in the alloy is above 0.45 wt% [24]. At higher Mn contents, deterioration of mechanical properties may occur due to the formation of a larger amount of intermetallic phases [25]. As with manganese, higher chromium contents deteriorate the properties of the alloys due to the formation of larger amounts of iron-containing phases [26,27,28]. Chromium is considered more effective when used in a ratio to iron (Cr:Fe) above 0.33 [22], and it has a positive effect on the properties of the alloy after heat treatment [19,29,30,31,32,33]. Cobalt is considered less effective than manganese and chromium, but it has a less harmful effect on the alloys. Although less frequently used, there is evidence of the positive modifying effect of molybdenum (Mo) on the AlFeSi phase [22].

To modify the eutectic in the composition of alloys, sodium (Na), strontium (Sr) and antimony (Sb) are usually used, which change the eutectic morphology to a finer fibrous structure [34]. The positive influence of sodium is discussed in [34,35,36], and the positive influence of strontium is discussed in [36,37,38,39,40]. The combination of Na and Sr leads to an increase in the modifying effect of the alloys [34]. Modification with antimony is characterized by the formation of lamellar eutectics [34,41], and its positive effect is presented in [19,41,42].

To modify the α-crystals in the alloy structures, titanium (Ti) and boron (B) introduced into the melts through Al-Ti-B-based ligatures are most often used [35].

For modifying free silicon crystals in the structures of hypereutectic compositions, phosphorus (P) has found the most widespread application [43,44,45] but other modifiers, including nanoscale ones, are also being experimented with [46,47].

The aim of this work is to investigate the influence of the alloying elements Ni, Co, Mo and the combinations thereof on the crystallization process, phase composition and corrosion resistance of the AlSi25Cu4Cr and AlSi25Cu5Cr alloys.

## 2. Materials and Methods

The object of the present study are the hypereutectic aluminum–silicon alloys AlSi25Cu4Cr and AlSi25Cu5Cr, which are additionally alloyed with the elements Ni, Co, Mo and their combinations. The alloys are melted in an electric resistance furnace under a layer of covering–refining flux (10 KCl:50 NaCl:10 Na_3_AlF_6_) in an amount of 0.5 wt% of the mass of the alloys. The AlSi25Cu4Cr alloy is additionally alloyed with both Ni, Cr and Mo, and with the combination of Ni, Cr, Co and Mo. The AlSi25Cu5Cr alloy is alloyed with the combination of the elements Co, Cr and Mo in different concentrations. For alloying the alloys with Ni, Co and Mo, and for introducing additional amounts of Cr into the composition of the base alloys, the dental alloys “wiron light” and “wironit” [48,49,50] were used, and the chemical composition of the two alloys is shown in Table 1 and Table 2.

Dental alloys were introduced into the melts of the studied alloys at a temperature of 810 °C. After alloying and mechanical stirring of the melts, they were kept for 30 min at a temperature increased to 850 °C. Metallurgical processing of the alloys was carried out, which included refining, degassing (argon purging) and modification with phosphorus. The compositions based on the AlSi25Cu4Cr alloy were modified with phosphorus in an amount of 0.04 wt% and those based on the AlSi25Cu5Cr alloy with phosphorus 0.07 wt%. Phosphorus was introduced into the melts by using the CuP10 ligature. The metallurgical processing of the melt was carried out at a temperature of 850 °C, and the casting temperature was 830 °C. The thermal analysis of the studied alloys was carried out using the specialized software PicoLog6, with data being recorded every 100 ms and temperature measurements being taken with a thermocouple type K (Ni-CrNi). Casting cores in the form of a cup were used for casting test bodies from both alloys, and the weight of the castings was about 0.6 kg. Experimental castings were cast to produce samples for conducting phase analysis and corrosion studies of the alloys using metal equipment preheated to 200 °C.

To determine the chemical composition of the studied alloys, a spectral analysis of the studied alloys was performed using an Oxford Instruments FOUNDRY-MASTER UV apparatus (Abingdon, UK). The FOUNDRY-MASTER UV is a reliable, precise laboratory spectrometer for the qualitative and quantitative element analysis of metallic samples. The instrument is designed for stationary use as a benchtop unit. The instrument is based on Optical Emission Spectroscopy (OES), the analyzing method favored by most metal-producing and metal-processing companies. The digital source (spark generator) is controlled via the external Windows^®^ PC and offers ideal excitation parameters for the most diverse alloys. The high-resolution Multi-CCD optics utilizes a traditional, robust, vacuum technology chamber rather than eccentric ways of removing harmful atmosphere from the optics. The optics covers the complete wavelength range from 160 nm to 800 nm.

After processing the results of the registered time–temperature curves, and especially the temperature of the solidus lines of the alloys, the operating parameters of the subsequent heat treatment were selected. The castings were heat treated according to the T6 regime, in which the heating for homogenization of the structure was at a temperature of 510–515 °C, the holding time at this temperature was 6 h 30 min, and the cooling medium used for quenching was water with a temperature of 20 °C. Artificial aging after quenching of the test bodies was carried out at a temperature of 180 °C, and the holding time at the selected operating temperature was 12 h.

A heat treatment phase analysis of the studied alloys was performed. The X’Pert PRO MPD diffractometer (Malvern Panalytical B.V., Almelo, The Netherlands) with cobalt radiation was used for obtaining X-ray diffraction (XRD) patterns. Phase analysis was performed in HighScore Plus (Malvern Panalytical B.V., Almelo, The Netherlands) using the PDF2 database. The irradiated volume was defined by the experimental geometry of the diffractometer (Bragg–Brentano), the effective penetration depth of the X-ray radiation (approx. 10 µm), and the pinhole size (1 × 1 mm). Diffraction data were obtained from a surface that had been previously electropolished to minimize the effect of surface preparation and thus to describe the bulk phase composition.

The microstructure of the alloys was studied using a Leica DM ILM microscope (Wetzlar, Germany) with the help of software and a module for grain measurement and phase analysis. The microsections were wet ground on sandpaper with the numbers 240, 320, 400, 600, 800 and 1000 and mechanically polished with diamond paste and lubricant. The microstructure of the samples was developed with Keller’s reagent (1 part HF, 1.5 parts HCl, 2.5 parts HNO_3_, 95 parts H_2_O).

After the alloys were subjected to T6 heat treatments, corrosion tests of the compositions were carried out in 1 M HCl solution and 1 M H_2_SO_4_ solution for 336 h and 504 h using the gravimetric method, in which the mass loss of the test bodies determines their corrosion rate. The total surface area of each of the samples is 0.02 m^2^. Before testing, the samples were placed in ethyl alcohol for 10 min, washed with distilled water, dried and weighed on an Acculab ATILON analytical balance. Then, they were immersed in 1 M H_2_SO_4_ and HCl solutions at room temperature. The measurements were made after the test bodies had been in the tested environments for 336 and 504 h. After each test period, samples of the tested alloys were cleaned with a brush under running water, dried and weighed, and the test solutions were replaced with new ones. For each of the periods of the sample study, the mass loss and corrosion rate (CR g/m^2^·h) were calculated and the research methodology is presented in [51].

## 3. Results

After casting the experimental castings, a spectral analysis of the studied alloys was carried out using an Oxford Instruments Foundry Master UV apparatus, and Table 3 shows the designations adopted for the present studies and the chemical composition of the alloys.

**Figure 1:**
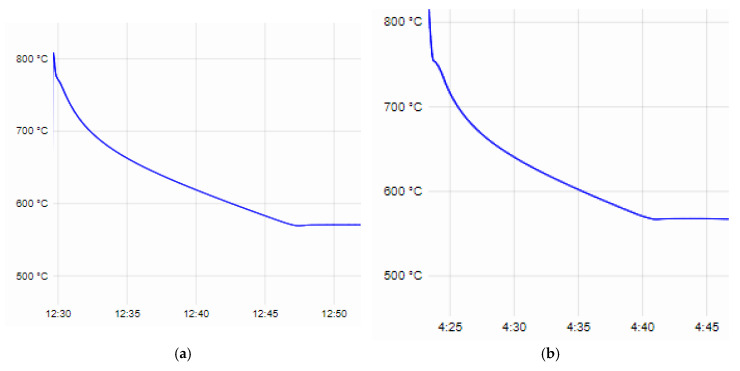

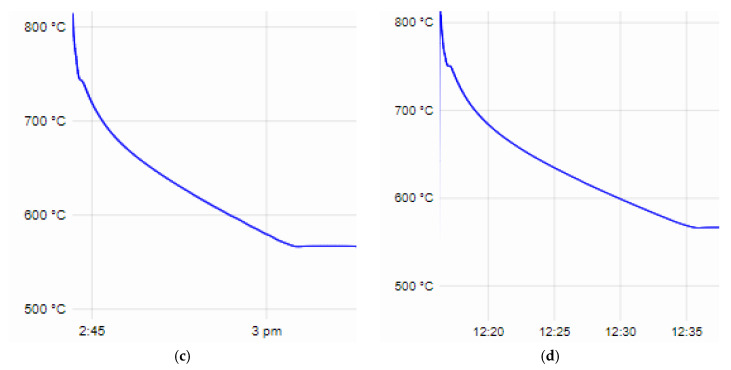
Time–temperature curves recorded during thermal analysis: (**a**) AlSi25Cu4Cr alloy; (**b**) AlSi25Cu4CrNiMo alloy (No. 1); (**c**) AlSi25Cu4CrNiCoMo alloy (No. 2); (**d**) AlSi25Cu4CrNiCoMo alloy (No. 3).

From the recorded time–temperature curves obtained during the thermal analysis, data were taken for the beginning and end of the crystallization process, the degree of supercooling at the eutectic crystallization temperature of both the base alloys AlSi25Cu4Cr and AlSi25Cu5Cr and the additionally alloyed compositions. The liquidus line temperature of the AlSi25Cu4Cr alloy is 776 °C, the solidus line is 569.5 °C and the degree of supercooling at the eutectic crystallization temperature is 1.2 °C (Figure 1a). The AlSi25Cu4Cr alloy additionally alloyed with Ni and Mo (No. 1) has a lower temperature in regard to the liquidus and solidus lines of the alloy (T_L_—755.8 °C and T_S_—567 °C), and the degree of supercooling at the solidus line is ∆T—0.7 °C (Figure 1b). In the case of the AlSi25Cu4Cr alloy additionally alloyed with Ni, Co and Mo (No. 2), a decrease in the studied parameters of the crystallization process was again registered (T_L_—753.8 °C, T_S_—566.3 °C, ∆T—0.6 °C), as seen in Figure 1c. In the case of alloy No. 3, the tendency to decrease the temperatures at the beginning and end of the crystallization process (T_L_—753.2 °C, T_S_—566 °C) is maintained as they are lower than those of the base alloy, and a small degree of supercooling (∆T—0.6 °C) was again registered during the eutectic crystallization of the alloy (Figure 1d). The alloying elements Ni, Co and Mo used in different concentrations narrow the temperature range of crystallization, but this does not lead to a change in the sizes of the primary silicon crystals in the structure of the alloys—they are comparable [52,53]. As a result of the smaller degree of supercooling at the eutectic crystallization temperature of alloys No. 1, No. 2 and No. 3 compared to the base alloy, the microstructural analysis found that the silicon crystals in the composition of the eutectic are larger [52,53]. It was found that, after the applied T6 heat treatment, the eutectic silicon crystals changed their shape (from lamellar to granular) and reduced their sizes [53].

**Figure 2:**
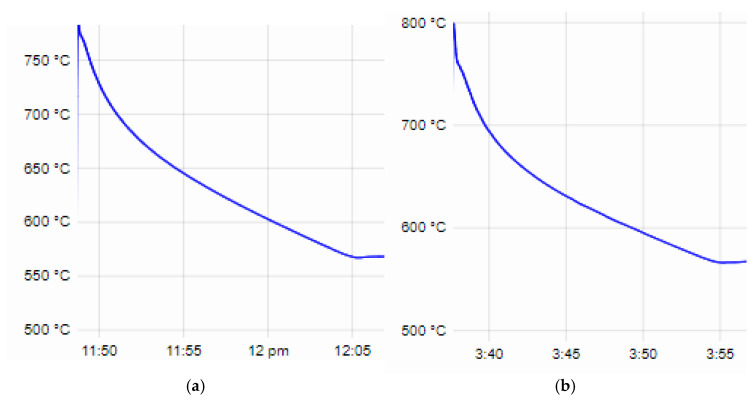

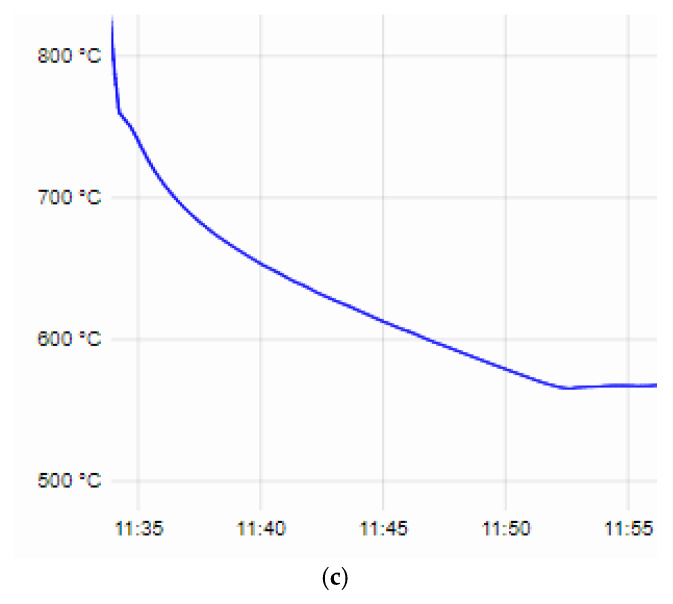
Time–temperature curves recorded during thermal analysis: (**a**) AlSi25Cu5Cr alloy; (**b**) AlSi25Cu4CrCoMo alloy (No. 4); (**c**) AlSi25Cu4CrNiCoMo alloy (No. 5).

The beginning of the crystallization process of the base alloy AlSi25Cu5Cr begins at a temperature of T_L_—779 °C and ends at a temperature of T_S_—567.6 °C. The supercooling of the alloy at the eutectic temperature is ∆T—1.3 °C (Figure 2a). Alloying the alloy with different concentrations of Co and Mo leads to a decrease in both the temperature of the liquidus line and the solidus line of the alloys, and no significant difference in the degree of supercooling of the alloys ∆T was recorded. The parameters of the crystallization process of alloy No. 4 are T_L_—759.3 °C, T_S_—566.7 °C and ∆T—1.3 °C (Figure 2b); for alloy No. 5, they are T_L_—758.8 °C, T_S_—566.5 °C and ∆T—1.1 °C (Figure 2c). The results of the microstructural analysis conducted show no differences in the structure of the base and those alloyed with Co and Mo, and, after T6 heat treatment, the eutectic silicon in the structure of the alloys has a spheroidal shape and small dimensions [52,53].

The values of the liquidus and solidus line temperatures, as well as the degrees of supercooling of the alloys at the eutectic crystallization temperature obtained from the thermal analysis, are presented in Table 4.

**Figure 3:**
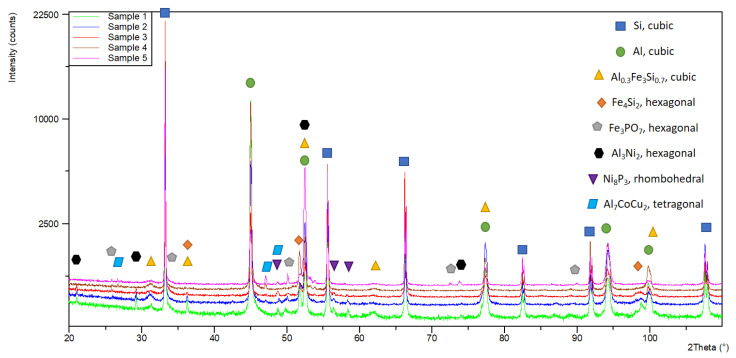
Comparison of diffraction diagrams of samples.

To study the influence of alloying elements on the structural composition of the alloys, a phase analysis was conducted. Three iron-containing phases were registered in the structure of the studied compositions. While the presence of the phases Al_0.3_Fe_3_Si_0.7_ and Fe_4_Si_2_ is expected, the compound Fe_3_PO_7_ in the composition of which phosphorus is involved is of interest. In alloys No. 1, No. 2 and No. 3, which are alloyed with Ni another phosphorus-containing phase, Ni_8_P_3_ was registered. The presence of intermetallics with the participation of phosphorus confirms the assumption that phosphorus works not only as a type II modifier but also as a surface-active substance (type I modifier) and participates in the composition of various chemical compounds, which, in turn, reduces its modifier effect on primary silicon crystals in the composition of hypereutectic aluminum–silicon alloys. In the composition of alloys alloyed with Ni, the Al_3_Ni_2_ phase is registered, which, according to a number of authors, is the basis of the increased heat resistance of aluminum alloys. Intermetallics with the participation of Cr and Mo are not registered, which in turn indicates that they participate in the α-solid solution. Alloying with minimal amounts of Co (alloys No. 2 and No. 3) does not lead to the formation of phases with the participation of cobalt; it, apparently, only dissolves in Al and strengthens the α-crystals. By increasing the amount of cobalt (alloys No. 4 and No. 5), a complex intermetallic with its participation (Al_7_CoCu_2_) is registered. The influence of alloying elements on the structure and mechanical properties of the studied alloys is discussed in [53]. The results of the phase analysis of the studied alloys are shown in Table 5, and Figure 3 shows an example comparison of samples from the study.

**Figure 4:**
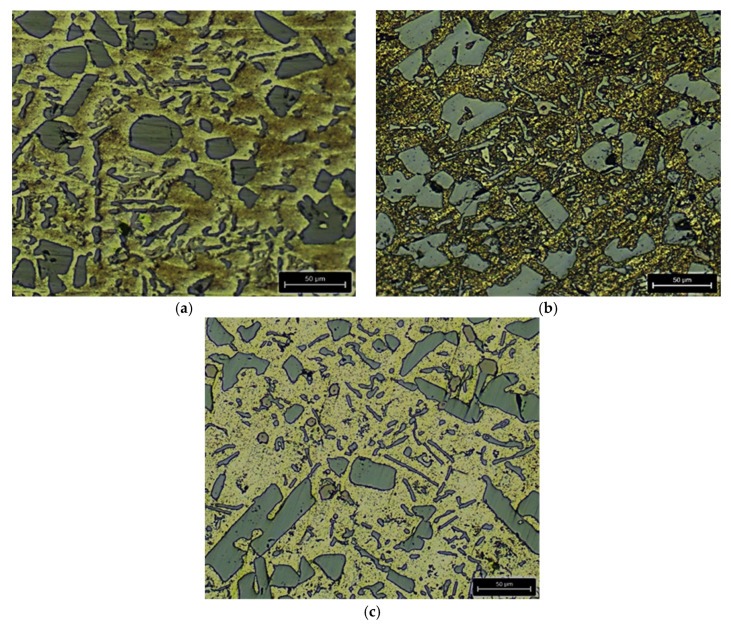

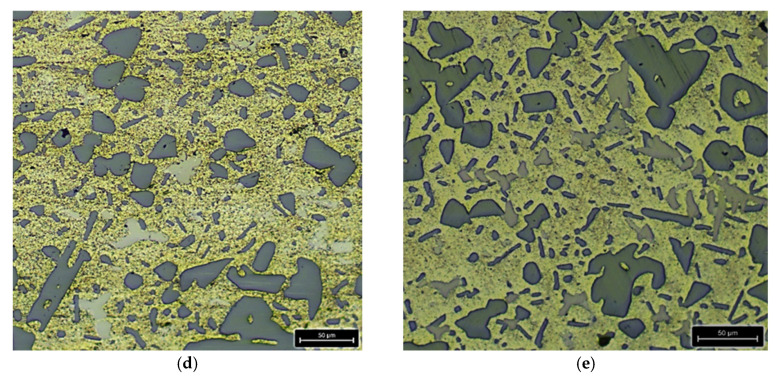
Structures of the studied alloys after T6 heat treatment: (**a**) AlSi25Cu4CrNiMo alloy (No. 1); (**b**) AlSi25Cu4CrNiCoMo alloy (No. 2); (**c**) AlSi25Cu4CrNiCoMo alloy (No. 3); (**d**) AlSi25Cu5CrCoMo alloy (No. 4); (**e**) AlSi25Cu5CrCoMo alloy (No. 5).

After T6 heat treatment, a microstructural analysis of the studied alloys was carried out. In the structure of alloy No. 1 (AlSi25Cu4CrNiMo), the silicon crystals in the eutectic composition are in the form of plates with a conditional average linear size of 13.15 µm. The free silicon crystals have a relatively regular shape and a conditional average diameter of 19.6 µm. Single silicon crystals with an irregular shape are also observed (Figure 4a). In the structure of alloy No. 2 (AlSi25Cu4CrNiCoMo), the silicon crystals can be conditionally divided into two groups. The first has a conditional average diameter of 16.4 µm, and the second has dimensions of about 40.5 µm. Single silicon crystals of irregular shape are also observed. Eutectic Si is in the form of plates with dimensions reaching 26.05 µm (Figure 4b). Similarly to alloy No. 2, in alloy No. 3 (AlSi25Cu4CrNiCoMo) the primary silicon crystals are of different sizes. One group of silicon crystals are of regular shape and sizes, up to 27.7 µm, and the second group of silicon crystals are of irregular shape and have a conditional average diameter of 52.5 µm. The eutectic silicon crystals are of needle-like shape, with an average linear size of 17.2 µm (Figure 4c). In the structure of alloy No. 4 (AlSi25Cu5CrCoMo), the free silicon crystals are relatively irregular in shape. The measured and calculated conditional mean diameter is 32.2 µm. Silicon in the composition of the eutectic is spheroidal in shape and has a conditional mean diameter of 11 µm (Figure 4d). The results of the microstructural analysis of alloy No. 5 (AlSi25Cu5CrCoMo) show that, as in alloy No. 4, the free silicon crystals are irregular in shape. They are small and have a conditional average diameter of 21.27 µm. According to their shape, the eutectic silicon in the structure of this alloy can be conditionally divided into two groups. The first group contains needle-shaped crystals with average linear dimensions of 15.2 µm, and the second group includes eutectic silicon crystals with a spheroidal shape and a conditional average diameter of 5.49 µm (Figure 4e). The results obtained from the microstructural analysis of the studied alloys confirm the assumption that the participation of the modifier phosphorus in various intermetallics (Fe_3_PO_7_, Ni_8_P_3_) reduces its modifying effect on the primary silicon crystals in the composition of the hypereutectic aluminum–silicon alloys.

To study the influence of the alloying elements Ni, Co and Mo after T6 heat treatment on the corrosion resistance of the alloys, corrosion tests of the compositions in 1 M HCl solution and 1 M H_2_SO_4_ solution for 336 h and 504 h were carried out. The gravimetric method was used, in which the mass loss of the test bodies determines their corrosion rate, and the research methodology is presented in [51]. The results of the calculated corrosion rate (CR g/m^2^·h) of the compositions in 1 M HCl are presented in Table 6, and the results obtained for the investigated characteristic (CR g/m^2^·h) in 1 M H_2_SO_4_ are shown in Table 7.

The use of combinations of alloying elements Ni and Mo, Ni, Co and Mo, as well as Co and Mo for alloying the AlSi25Cu4Cr and AlSi25Cu5Cr alloys, is appropriate. In the case of AlSi25Cu4Cr and AlSi25Cu5Cr, alloys alloyed with refractory metals (Ni, Co and Mo) over the course of the study, it was observed, with an increase in the concentration of the alloying elements, their corrosion resistance in 1 M H_2_SO_4_ increases. The use of the alloying elements Ni, Co and Mo has a positive effect on the corrosion resistance of the AlSi25Cu4Cr and AlSi25Cu5Cr alloys in 1 M HCl, and the same tendency is observed in them during boiling as in 1 M H_2_SO_4_. The mass loss of the test specimens in 1 M HCl solution is lower compared to 1 M H_2_SO_4_ solution, i.e., under the specified experimental conditions, the alloys have a low corrosion rate in 1 M HCl.

On the surface of aluminum and its alloys in acidic media, there are usually different areas on which anodic and cathodic reactions are localized, represented by the following equations:

Anodic Reaction: 2Al → 2Al^3+^ + 6e^−^

Cathodic Reaction: 6H^+^ + 6e^−^ → 3H_2_

In the samples subjected to longer exposure in 1 M H_2_SO_4_, a lower corrosion rate was observed (Table 7, samples 1, 3, 4 and 5), with the probable reason for this being the formation of a protective layer on the surface of the studied alloys.

**Figure 5:**
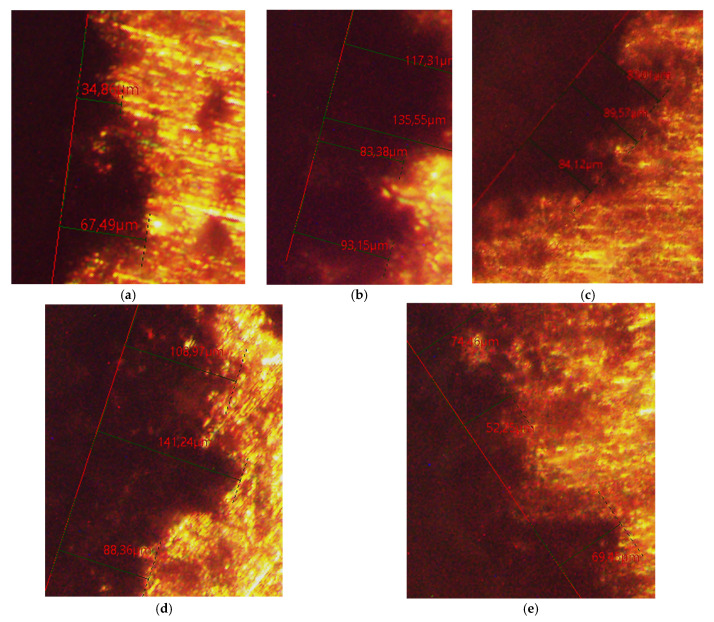
Maximum measured penetration depth of the corrosive medium 1 M H_2_SO_4_ after the maximum test period of 504 h in the test specimens of the tested alloys: (**a**) AlSi25Cu4CrNiMo alloy (No. 1); (**b**) AlSi25Cu4CrNiCoMo alloy (No. 2); (**c**) AlSi25Cu4CrNiCoMo alloy (No. 3); (**d**) AlSi25Cu5CrCoMo alloy (No. 4); (**e**) AlSi25Cu5CrCoMo alloy (No. 5).

**Figure 6:**
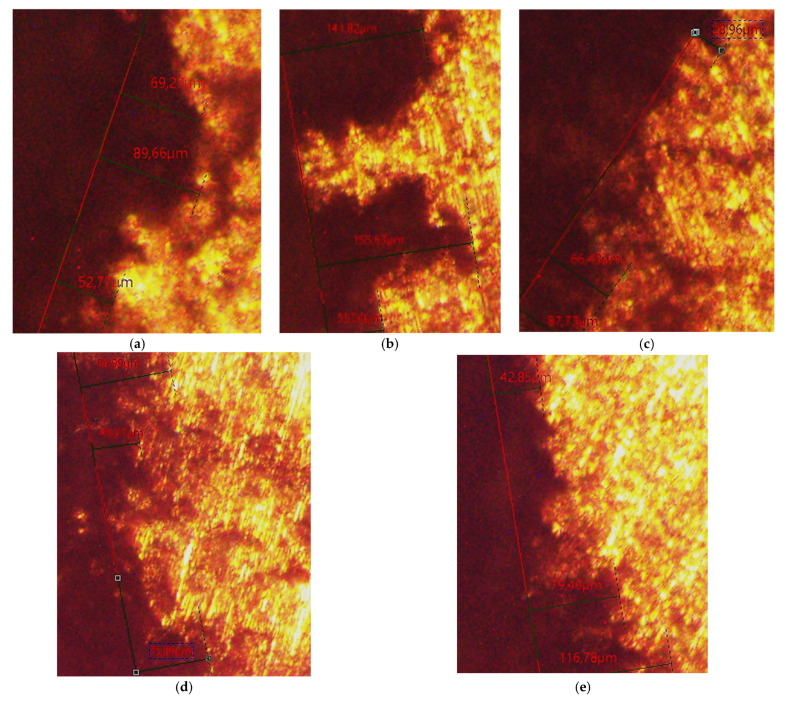
Maximum measured penetration depth of the corrosive medium 1 M HCl after the maximum test period of 504 h in the test specimens of the tested alloys: (**a**) AlSi25Cu4CrNiMo alloy (No. 1); (**b**) AlSi25Cu4CrNiCoMo alloy (No. 2); (**c**) AlSi25Cu4CrNiCoMo alloy (No. 3); (**d**) AlSi25Cu5CrCoMo alloy (No. 4); (**e**) AlSi25Cu5CrCoMo alloy (No. 5).

Using a Leica DM ILM metallographic microscope, the maximum penetration depth of the used corrosion media (1 M H_2_SO_4_ and 1 M HCl) in the test specimens of the studied alloys after the maximum test period (504 h) was measured. Figure 5 shows the results of the test in 1 M H_2_SO_4_, and Figure 6 shows the results of the test in 1 M HCl.

## 4. Discussion

The conducted studies have established the influence of the combinations of alloying elements Ni, Co and Mo on the crystallization process of non-standardized hypereutectic aluminum–silicon alloys. The temperature range of the studied compositions is narrow, but nevertheless, no differences in the shape and size of the primary silicon crystals in the alloy structures are observed [52,53]. The narrower temperature range of crystallization is a prerequisite for the reduction in micro-suction porosity during solidification of the castings, which will inevitably lead to an increase in their quality. The temperatures at which the crystallization process begins have been registered, which is of essential importance for the technological process of melting and the casting of the alloys. Knowing the liquidus temperatures of the alloys, there is no possibility of excessive overheating of the melts or a risk of their gas saturation. It is possible to determine the optimal casting temperature of the alloys, which will avoid the state of zero thinness, i.e., to obtain castings with exact shapes and dimensions. The temperatures of eutectic crystallization of the studied compositions have been taken into account. This is of extreme importance in regard to the subsequent heat treatment of the alloys. The determination of the temperature for heating and homogenizing the structure of the alloys for quenching is directly dependent on the temperature of the solidus lines of the alloys. The correct choice of the heating temperature before quenching is a prerequisite of the maximum dissolution of the alloying elements in the α-crystals and the fixation of a maximally saturated α-solid solution after quenching. Thus, during the subsequent artificial aging, the process of formation, growth and distribution of secondary strengthening phases can be controlled, i.e., alloys with increased mechanical and improved operational properties can be obtained. The alloying elements used and their combinations are the basis for obtaining alloys with increased mechanical properties in tests conducted at normal temperatures [53]. During the phase analysis, it was established that the alloying elements Cr and Mo do not form strengthening phase, and it was also established that they participate in the α-solid solution. The same applies to Co when it is used in minimal quantities; when using higher concentrations of Co, its participation in the ternary intermetallic Al_7_CoCu_2_ (alloys No. 4 and No. 5) is registered. The presence of the phosphorus-containing phases Fe_3_PO_7_ and Ni_8_P_3_ confirms the assumptions that phosphorus works not only as a type II modifier but also as a surfactant (type I modifier); it also participates in the composition of various chemical compounds, which, in turn, reduces its modifying effect on primary silicon crystals in the composition of hypereutectic aluminum–silicon alloys.

The use of combinations of the alloying elements Ni, Co and Mo has a more positive effect on the structure and properties of the AlSi25Cu4Cr and AlSi25Cu5Cr alloys [53] and makes them competitive with the established piston hypereutectic aluminum–silicon alloys. This, in turn, is a prerequisite for future studies of such alloyed alloys and will help in determining their mechanical properties at elevated temperatures and their wear resistance in tribological systems.

## 5. Conclusions

It was found that the combinations of the used alloying elements Ni, Co and Mo narrow the temperature range in regard to the crystallization of the studied alloys. The temperatures at the beginning and end of the crystallization process of the compositions were recorded, which is of utmost importance for the processes of melting and casting of the alloys, as well as for the subsequent heat treatment.

The elements Cr and Mo, as well as Co, when used in minimal amounts, do not form secondary strengthening phases but dissolve in the α-solid solution. The results of the corrosion tests conducted in 1 M HCl solution and 1 M H_2_SO_4_ solution for 336 h and 504 h show that the elements Ni, Co and Mo improve the corrosion resistance of the alloys compared to the base alloys AlSi25Cu4Cr and AlSi25Cu5Cr.

## Figures and Tables

**Table 1:** Chemical composition of “wiron light” alloy [wt%].

Alloy	Ni	Cr	Mo	Si
wiron light	64.6	22	10	2.1

**Table 2:** Chemical composition of “wironit“ alloy [wt%].

Alloy	Co	Cr	Mo	Mn	Si
wironit	64	28.5	5	1	1

**Table 3:** Designations and chemical composition of the studied alloys [wt%].

No.	Alloy	Si	Cu	Cr	Ni	Co	Mo	Fe	Al
1	AlSi25Cu4CrNiMo	24.01	3.73	0.633	0.752	-	0.114	0.298	rest
2	AlSi25Cu4CrNiCoMo	24.30	3.9	0.724	0.698	0.086	0.116	0.298	rest
3	AlSi25Cu4CrNiCoMo	24.18	3.98	0.742	0.733	0.112	0.12	0.243	rest
4	AlSi25Cu5CrCoMo	25.14	4.16	0.738	-	0.438	0.035	0.476	rest
5	AlSi25Cu5CrCoMo	25.14	4.05	0.810	-	0.595	0.05	0.5	rest

**Table 4:** Thermal analysis results.

No.	Alloy	T_L_, [°C]	T_S_, [°C]	∆T, [°C]
	AlSi25Cu4Cr	776 °C	569.5 °C	1.2 °C
1	AlSi25Cu4CrNiMo	755.8 °C	567 °C	0.7 °C
2	AlSi25Cu4CrNiCoMo	753.8 °C	566.3 °C	0.6 °C
3	AlSi25Cu4CrNiCoMo	753.2 °C	566 °C	0.6 °C
	AlSi25Cu5Cr	779 °C	567.6 °C	1.3 °C
4	AlSi25Cu5CrCoMo	759.3 °C	566.7 °C	1.3 °C
5	AlSi25Cu5CrCoMo	758.8 °C	566.5 °C	1.1 °C

**Table 5:** Phase analysis results.

Phase	Alloy				
	No. 1	No. 2	No. 3	No. 4	No. 5
Si, cubic	✓	✓	✓	✓	✓
Al, cubic	✓	✓	✓	✓	✓
Al_0.3_Fe_3_Si_0.7_, cubic	✓	✓	✓	✓	✓
Fe_4_Si_2_, hexagonal	✓	✓	✓	✓	✓
Fe_3_PO_7_, hexagonal	✓	✓	✓	✓	✓
Al_3_Ni_2_, hexagonal	✓	✓	✓		
Ni_8_P_3_, rhombohedral	✓	✓	✓		
Al_7_CoCu_2_, tetragonal				✓	✓

**Table 6:** Corrosion rate (CR g/m^2^·h) in 1 M HCl.

No.	Alloy	336 h	504 h
1	AlSi25Cu4CrNiMo	0.0033	0.0036
2	AlSi25Cu4CrNiCoMo	0.0047	0.0051
3	AlSi25Cu4CrNiCoMo	0.0044	0.0045
4	AlSi25Cu5CrCoMo	0.0028	0.006
5	AlSi25Cu5CrCoMo	0.0036	0.0038

**Table 7:** Corrosion rate (CR g/m^2^·h) in 1 M H_2_SO_4._

No.	Alloy	336 h	504 h
1	AlSi25Cu4CrNiMo	0.0086	0.0077
2	AlSi25Cu4CrNiCoMo	0.01	0.0168
3	AlSi25Cu4CrNiCoMo	0.01	0.0093
4	AlSi25Cu5CrCoMo	0.0118	0.0112
5	AlSi25Cu5CrCoMo	0.0105	0.0102

## Data Availability

The original contributions presented in this study are included in the article. Further inquiries can be directed to the corresponding authors.
